# Technological, nutritional, and biological properties of apricot kernel protein hydrolyzates affected by various commercial proteases

**DOI:** 10.1002/fsn3.3467

**Published:** 2023-06-01

**Authors:** Khashayar Sarabandi, Maryam Mohammadi, Zahra Akbarbaglu, Marjan Ghorbani, Shahla Najafi, Sara Safaeian Laein, Seid Mahdi Jafari

**Affiliations:** ^1^ Department of Food Science & Technology, School of Medicine Zahedan University of Medical Sciences Zahedan Iran; ^2^ Department of Food Science and Engineering, Faculty of Agriculture University of Kurdistan Sanandaj Iran; ^3^ Drug Applied Research Center Tabriz University of Medical Sciences Tabriz Iran; ^4^ Department of Food Science, College of Agriculture University of Tabriz Tabriz Iran; ^5^ Nutrition Research Center Tabriz University of Medical Sciences Tabriz Iran; ^6^ Department of Biology, Faculty of Science University of Zabol Zabul Iran; ^7^ Department of Food Hygiene and Aquaculture, Faculty of Veterinary Medicine Ferdowsi University of Mashhad Mashhad Iran; ^8^ Department of Food Materials & Process Design Engineering Gorgan University of Agricultural Sciences and Natural Resources Gorgan Iran

**Keywords:** antioxidant properties, apricot kernel protein, enzymatic hydrolysis, hydrolyzates

## Abstract

The effect of enzymatic hydrolysis of apricot kernel protein with different proteases (Alcalase, pancreatin, pepsin, and trypsin) on the amino acid content, degree of hydrolysis (DH), antioxidant, and antibacterial characteristics of the resulting hydrolyzates was investigated in this study. The composition of amino acids (hydrophobic: ~35%; antioxidant: ~13%), EAA/TAA ratio (~34%), and PER index (~1.85) indicates the ability of the hydrolyzate as a source of nutrients and antioxidants with high digestibility. Enzymatic hydrolysis with increasing DH (from 3.1 to a maximum of 37.9%) led to improved solubility (especially in the isoelectric range) and changes in water‐ and oil‐holding capacity. The highest free radical scavenging activity of DPPH (83.3%), ABTS (88.1%), TEAC (2.38 mM), OH (72.5%), NO (65.7%), antioxidant activity in emulsion and formation of TBARS (0.36 mg MDA/L), total antioxidant (1.61), reducing power (1.17), chelation of iron (87.7%), copper (34.8%) ions, and inhibition of the growth of *Escherichia coli* (16.3 mm) and *Bacillus cereus* (15.4 mm) were affected by the type of enzymes (especially Alcalase). This research showed that apricot kernel hydrolyzate could serve as a nutrient source, emulsifier, stabilizer, antioxidant, and natural antibacterial agent in functional food formulations.

## Graphical abstract

Technological, nutritional, and biological properties of apricot kernel protein hydrolyzates are affected by various commercial proteases.

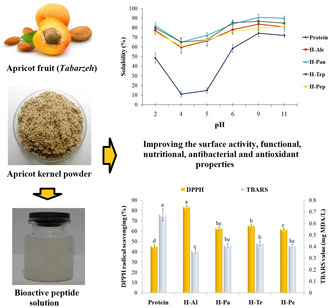

## 1 INTRODUCTION

Agro‐industrial wastes such as fruit seeds are mainly considered as waste, which causes environmental pollution and must be discarded; however, they can be converted to value‐added products with high protein content and high nutritional values in the food industry (Rammuni et al., 2019; Tan et al., 2019). As the world population continues to steadily grow, the global demand for dietary protein sources is rapidly increasing. In recent years, there has been a growing interest in enzymatic hydrolysis of protein‐rich by‐products to obtain peptides with techno‐functional and nutritional properties. Protease enzymes from various sources can cleave peptide bonds, resulting in the production of soluble hydrolyzates with significant antioxidant activity that can reduce lipid peroxidation and oxidative stress (Clerici et al., 2021).

The amino acid sequences of peptides can be different depending on substrate type, enzyme type, specificity, degree of hydrolysis (DH), and others (Bozkurt et al., 2021). DH is an important factor representing hydrolysis efficacy and is closely associated with the molecular weight distribution of resulting hydrolyzates (Zheng et al., 2019). Lipid oxidation caused by free radicals and microbial spoilage are the key factors that influence the shelf life and quality of food products (Calderón‐Chiu et al., 2021; Lima et al., 2019). To maintain the quality of food, synthetic preservatives and antioxidant additives are commonly used in the food industry, but they may have potential toxicity to consumers' health, leading to restrictions on their use. Therefore, there is a growing need to find natural preservative and antioxidant compounds that can replace synthetic ones, which is an important issue (Jang et al., 2016). On the other hand, consumer demand for safe, natural, and functional products has increased. Some peptides and protein hydrolyzates are identified to have antioxidant activity. For example, peptides extracted from hen egg white (Memarpoor‐Yazdi et al., 2012), fish skin gelatin (Mendis et al., 2005), Alaskan Pollack hydrolyzates (Je et al., 2005), *Spirulina* algae protein (Akbarbaglu et al., 2022; Mohammadi et al., 2022), black bean protein (Zheng et al., 2019), and flaxseed protein (Sarabandi & Jafari, 2020a) exert antioxidant effects. Other health‐promoting effects and biological properties of protein hydrolyzates include antibacterial, antihypertensive, hypocholesterolemic, opioid, immunomodulatory, and antidiabetic activities (Marciniak et al., 2018).

Apricot kernel (*Prunus armeniaca*) is rich in protein (viz., albumin, globulin, glutelin, and prolamin), fatty acids, dietary fibers, minerals, vitamins, and phenolic compounds. These chemicals can play a significant role in preserving health and avoiding numerous disorders (Huang et al., 2022). A significant part of the annual production of apricots (>4 million tons) is made up of their core and shell. The richness of apricot kernel in protein (15%–45%) makes it a potential source of production of bioactive hydrolyzates with functional features (Akhone et al., 2022; Rampáčková et al., 2021). Thus far, no comprehensive study has been conducted on the generation of bioactive hydrolyzates derived from various proteases, as well as the assessment of the nutritional, functional, antioxidant, and antibacterial characteristics of apricot kernels.

Therefore, our objective was to suggest a natural, nutritious, and reliable source of by‐product materials such as apricot kernel hydrolyzates as an antioxidant agent instead of synthetic ones for application in the food industry. In this regard, the effect of proteolysis of apricot kernel protein (AKP) with different proteases (pepsin, pancreatin, trypsin, and Alcalase) on the nutritional and techno‐functional of apricot kernel peptides was investigated. Because the antioxidant activity of hydrolyzates is influenced by a variety of factors such as DH, molecular weight, and amino acid sequence, a variety of techniques such as DPPH radical scavenging, TEAC, hydroxyl radical scavenging, reducing power, nitric oxide scavenging, total antioxidant, and chelation of iron and copper ions are used to evaluate the antioxidant activity of hydrolyzates. Finally, the antibacterial activity of the hydrolyzates against food‐borne pathogens (*Escherichia coli* and *Bacillus cereus*) was investigated.

## 2 MATERIALS AND METHODS

### 2.1 Materials

The chemicals containing ABTS (2,2′‐azino‐bis (3‐ethylbenzothiazoline‐6‐sulfonic acid) diammonium salt), Alcalase 2.4 L (Novo Nordisk), comasi brilliant blue (G250), ferrozine (3‐(2‐pyridyl)‐5‐6‐diphenyl‐1,2,4‐triazine‐4′,4″ disulfonic‐acid sodium salt), DPPH (1,1‐diphenyl‐2‐picrylhydrazyl), pepsin (porcine gastric mucosa), pancreatin from porcine pancreas (P1750, 4 × USP specifications), sodium nitroprusside (SNP), sulfanilamide (SA), trypsin (bovine pancreas), naphthylethylenediamine dihydrochloride (NEDD), Trolox (6‐hydroxy‐2,5,7,8‐tetramethychroman‐2‐carboxylic acid), and pyrocatechol violet were purchased from Merck. Fluka contributed the alpha‐deoxyribose. Potassium persulphate, trichloroacetic acid (TCA), thiobarbituric acid (TBA), iron (II) chloride, and other materials were obtained from Merck.

### 2.2 Protein extraction

Apricot fruits (CV *Tabarzeh*) were obtained from the orchards of East Azerbaijan, the kernels were broken, and the seeds were separated and ground. Using the procedure described by Sarabandi and Jafari (2020b), we prepared and extracted the protein concentrate from the apricot kernel flour. In brief, the oil separation from the flour was carried out by a Soxhlet extractor. After that, the oil‐free flour was solubilized in the 0.1% salty solution with pH: 9.5 for 1 h, followed by centrifugation at 7000 *g* for 20 min. The soluble protein was then precipitated by adjusting the pH to 4.2 (isoelectric pH) with 0.5 M HCl, followed by centrifugation at 7000 *g* for 20 min. Lastly, the pH of the pellet was adjusted to 7 using 0.5 M NaOH, and freeze‐drying of the solution was carried out (Christ).

### 2.3 Protein hydrolyzate preparation

The powdered AKP was solubilized in a 5% w/v solution of 0.01 M phosphate‐buffered saline (PBS) for 30 min at 50°C. The hydrolysis process was then implemented using Alcalase (pH = 8, 50°C), pancreatin (pH = 8, 37°C), pepsin (pH = 2, 37°С), and trypsin (pH = 8, 37°C) at an enzyme/substrate ratio of 2% w/w for 30–180 min. The enzyme activity was stopped by incubating the reaction medium in a water bath at 95°C for 15 min, followed by centrifugation of the dispersion at 7000 *g* for 20 min. The supernatant containing the hydrolyzates was collected, freeze‐dried (using a Christ, freeze dryer), and then further analyzed (Akbarbaglu et al., 2022).

### 2.4 Degree of hydrolysis

In the same proportion, APK hydrolyzates (AKPH) and 0.44 M TCA were combined, incubated at 4°C for 10 min, and then centrifuged at 4000 *g* for 10 min. The soluble protein content was determined by the Bradford method (Bradford, 1976). The DH was calculated by Equation (1):

(1) $$\mathrm{DH}\;\left(\%\right)=\text{Protein}\;\left(\text{Supernatant}+\mathrm{TCA}\right)/\text{Total protein in the hydrolyzates}\times 100$$

### 2.5 Techno‐functional properties

Protein solubility was determined according to the method described earlier by Jamdar et al. (2010). Two‐hundred milligram of the samples was dissolved in 20 mL of distilled water. The pH of the solution was adjusted from 2 to 11 using 1–6 N HCl or NaOH. The solution was then centrifuged at 7000 × g for 10 min. The protein content in the supernatant was determined using a Bradford assay (Bradford, 1976). According to the method given by Ge et al. (2021), the water‐holding capacity (WHC) and oil‐holding capacity (OHC) of crude protein and hydrolyzates were determined. In brief, 1 g of each sample was mixed with 5 g of distilled water/soybean oil, mixing for 30 min, and then the mixture was centrifuged at 4000 *g* for 20 min. The supernatant was discarded and the pellet was weighed. The WHC and OHC were determined as grams of water/oil held by 1 g of the hydrolyzate.

### 2.6 Nutritional and amino acid composition

The samples were hydrolyzed with 6 N HCl at 110°C for 24 h and the effect of the hydrolysis process on the nutritional values and sequence of the amino acids was studied using a reversed‐phase HPLC (Novapack C18, 4 μm; Waters). Finally, the amino acid composition was expressed as mg/g dry matter (You et al., 2009). Also, protein efficiency ratios (PER) were calculated according to the following equation (Ge et al., 2021):

(2) PER=−0.468+0.454·Leu−0.105·Tyr

### 2.7 Antioxidant characterization

#### 2.7.1 DPPH free radical scavenging

AKP and its hydrolyzates (40 mg/mL) were mixed with 0.2 mM DPPH solution in the same ratio, and the mixture was then kept in a dark place for 30 min. After that, the mixture was centrifuged (5000 *g*, 10 min) and the absorbance of the supernatant was measured at 517 nm (Kimatu et al., 2017). The DPPH free radical scavenging was calculated according to the following equation:

(3) $$\text{Inhibition}\;\left(\%\right)=\left[1-\left({\text{sample}}_{\mathrm{Abs}}/{\text{blank}}_{\mathrm{Abs}}\right)\right]\times 100$$

#### 2.7.2 Antioxidant activity in emulsion

The ability of the crude protein and its hydrolyzates to preserve the oil/water emulsion against oxidation was determined by the technique reported by Abeyrathne et al. (2016). The procedure involved producing an O/W emulsion by emulsifying 1 g of grape‐seed oil with 100 μL of Tween‐20 into distilled water (100 mL) at 18,000 *g* for 2 min in an ice bath using a homogenizer. Next, for lipid oxidation evaluation, 8 mL of the prepared emulsion, 0.5 mL of vitamin C (0.2% w/v), 0.5 mL of ferrous sulfate (0.02% w/v), and 1 mL of hydrolyzates (40 mg/mL) were mixed, followed by incubating the samples at 37°C for 16 h. After the incubation time, 1 mL of the incubated samples, 2 mL of TBA/TCA solution (20 mM TBA/15% TCA), and 50 μL of 10% BHA dissolved in 90% ethyl alcohol were mixed by mechanical stirring. The mixture was then incubated in a water bath at 90°C for 15 min to improve color, and the color intensity of the cooled sample was recorded at 532 nm. The TBARS value was expressed as mg of malondialdehyde (MDA) per liter of emulsion.

#### 2.7.3 ABTS free radical scavenging

By combining the ABTS with potassium persulfate at a concentration of 2.45 mM and storing the mixture in the dark for 12 h at a concentration of 7 mM, ABTS free radical scavenging solution was made, which was then diluted in PBS buffer until reaching 0.70 absorbance at 734 nm. After that, 40 μL of the hydrolyzates (20 mg/mL) was mixed into 4 mL of the ABTS solution, vortexed for 20 s, and eventually incubated in dark for 6 min. Finally, the absorbance was recorded at 734 nm. To determine the TEAC, a standard curve was plotted by reacting the ABTS solution with various concentrations of Trolox (50–1000 μM; Phongthai et al., 2018).

#### 2.7.4 Hydroxyl radical scavenging

In this test, 0.5 mL of 0.01 M α‐deoxyribose, 0.9 mL of PBS buffer (0.2 M, pH = 7.4), 0.2 mL of 0.01 M FeSO_4_‐EDTA, 0.2 mL of hydrolyzate solution (40 mg/mL), and 0.2 mL of 0.01 M H_2_O_2_ were kept at 37°C for 60 min. After that, 1.0 mL of 1% TBA and 1.0 mL of 3% TCA were added to the reaction mixture, and it was kept in a boiling water bath for 15 min. Once the reaction mixture cooled down, the absorbance of samples was read at 532 nm (You et al., 2009).

#### 2.7.5 Reducing power

0.5 mL of a hydrolyzate solution (40 mg/mL) was added to a reaction mixture consisting of 0.5 mL of phosphate buffer (200 mM, pH = 6.6) and 0.5 mL of 1% potassium ferricyanide, and then incubated at 50°C for 20 min. The reaction mixture was then treated with 0.5 mL of 10% TCA solution and centrifuged at 3000 *g* for 10 min. After diluting 1 mL of the supernatant with 1 mL of purified water and 0.2 mL of 0.1% FeCl2, the absorbance at 700 nm was measured (Moghadam et al., 2020).

#### 2.7.6 Total antioxidant activity

The TAA was determined as described by Akbarbaglu et al. (2022). In brief, 0.2 mL hydrolyzate solution (20 mg/mL) was added to 2 mL of a reagent containing 0.028 M sodium phosphate, 0.6 M sulfuric acid, and 0.004 M ammonium molybdate, and the mixture was incubated in a boiling water bath for 90 min. After cooling, the absorbance of the mixture was monitored at 695 nm.

#### 2.7.7 Nitric oxide scavenging

In this method, 0.2 mL hydrolyzate solution (40 mg/mL) was mixed with 0.2 mL SNP solution (0.01 M) and incubated for 150 min in visible light. After that, the incubated mixture and newly produced Griess reagent (composed of an equal quantity of 1% SA and 0.1% NEDD in 2.5% phosphoric acid) were mixed with equal volumes, and the absorbance was recorded at 546 nm (Tsai et al., 2007).

#### 2.7.8 Fe^2+^‐chelating activity

At first, 1 mL of hydrolyzate solution (40 mg/mL) was added to the reaction mixture composed of 0.05 mL of FeCl_2_ solution (0.002 M), 1.8 mL of deionized water, and 0.1 mL Ferrozine solution (0.005 M) was vortex‐mixed and incubated at ambient temperature for 10 min. After that, the solution was read at 562 nm (Kimatu et al., 2017).

#### 2.7.9 Cu^2+^‐chelating activity

Initially, equal volumes of hydrolyzate solution (40 mg/mL) and copper sulfate (0.2 mM) were mixed and incubated at room temperature for 5 min. After that, 1 mL of 10% TCA solution was added to the mixture, followed by centrifugation at 2000 *g* for 10 min. Then, pyridine solution (1 mL, 10% w/v) and pyrocatechol violet solution (0.02 mL, 0.1% w/v) were added to 2 mL of the supernatant. The reaction mixture was vortexed and kept at room temperature for 5 min, and the absorbance was measured at 632 nm (Akbarbaglu et al., 2019).

#### 2.7.10 Antibacterial activity of hydrolyzate

The antibacterial activity of the hydrolyzates was examined by the agar well‐diffusion technique (Balouiri et al., 2016). In this method, 0.1 mL of overnight‐cultured bacteria (*E. coli O157:H7* and *B. cereus*) with a turbidity of 0.5 McFarland was spread uniformly on the entire agar medium. Then, 0.2 mL of the hydrolyzates (50 mg/mL) was poured into a well created by a sterile cork borer, and the plates were incubated at 37°C for 24 h (*E. coli*) and 28°C for 24 h (*B. cereus*), and the inhibitory area around each well was evaluated. Positive and negative controls were conducted using distilled water and oxytetracycline (0.05 mL per well), respectively.

### 2.8 Statistical analysis

The statistical analysis was performed using SPSS version 16.0 (SPSS Inc.) and involved one‐way analysis of variance (ANOVA). Mean values of treatments were compared using Duncan's test at a significance level of 5%.

## 3 RESULTS AND DISCUSSION

### 3.1 Degree of hydrolysis

DH is an important factor to evaluate the hydrolysis progress, which depends on the number of peptide bonds available for protease hydrolysis and the active site of protease that is exclusive to amino acid composition and protease origin (animal, plant, or microbial origin; Kristoffersen et al., 2020). The amount of DH for the AKP treated with pepsin (28.3%), pancreatin (35.5%), trypsin (37.9%), and Alcalase (32.8%) was affected by the type of enzyme. The cause of these findings can be attributed to the difference in specific function and proteolytic activity of enzymes (Lima et al., 2019). In another study, the DH of black bean protein after 300 min of digestion with Alcalase, bromelain, and ficin enzymes was found to be about 19%, 23%, and 7%, respectively (Zheng et al., 2019).

### 3.2 Solubility

**FIGURE 1:**
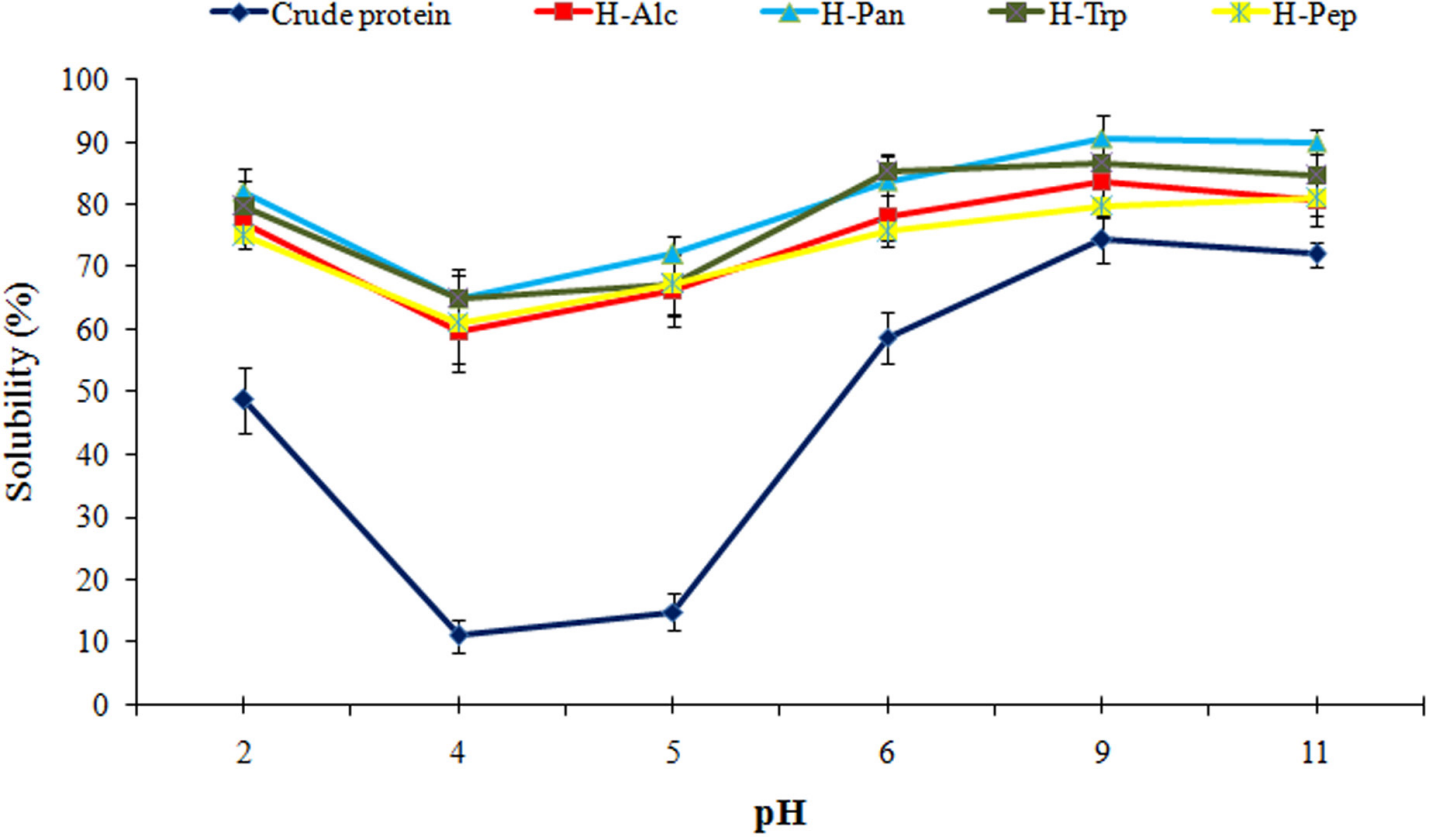
Effects of different enzymes and pH values on the solubility of apricot kernel protein. Alc, Alcalase; H, hydrolyzate; Pan, pancreatin; Pep, pepsin; Trp, trypsin.

The solubility profiles of AKP and its hydrolyzates are given in Figure 1. The solubility of the protein isolate was lower at isoelectric pH (pH = 4, 10%), which a given protein shows no net charge because the number of negative charges and positive ones is equalized, and the protein precipitates. From pH = 4.0 to 11.0, the solubility increased significantly (*p <* .05). This may be attributed to the increased negative charge of the protein as the pH move away from the isoelectric point. The solubility of the hydrolyzate was significantly greater (*p <* .05) than the crude protein's overall pH ranges. This indicated that the hydrolysis process enhanced the solubility of the protein even at acidic pH values (>80%) and isoelectric pH (>60%). This improved solubility of protein could simplify its insertion into acidic formulations such as fruit juices. The H‐Pan and H‐Try showed the highest solubility results, which can be attributed to their higher DH, and consequently, the lower‐molecular‐weight hydrolyzate compared to the other hydrolyzates. Several previous studies have also reported an increase in protein solubility as a result of the hydrolysis process (Ai et al., 2019; Klost & Drusch, 2019; Xu et al., 2016).

### 3.3 WHC and OHC

**FIGURE 2:**
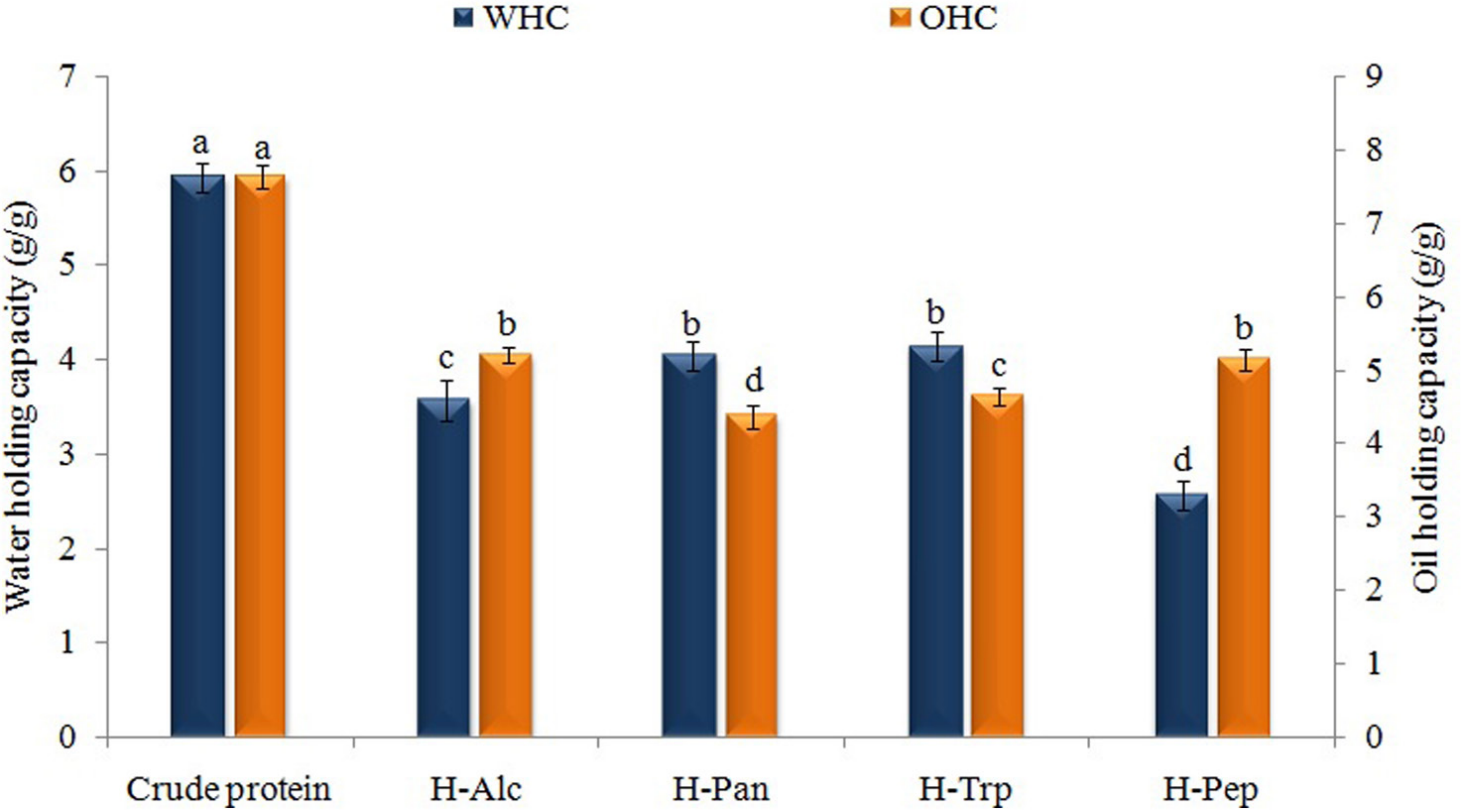
Effects of different enzymes on the water‐ (WHC) and oil (OHC)‐holding capacity of apricot kernel protein. Alc, Alcalase; H, hydrolyzate; Pan, pancreatin; Pep, pepsin; Trp, trypsin.

These important functional properties of proteins in the food industry demonstrate the potential of proteins or hydrolyzates to interact with water or lipids. Additionally, the W/O‐HC is expressed as the amount of water or oil absorbed by the protein or hydrolyzate (Ozorio et al., 2019). As can be seen from the results, the crude protein exhibited the highest W/O‐HC, and this value decreased significantly after hydrolysis by all enzymes (Figure 2). This decrease may be attributed to excessive proteolysis of proteins, which can reduce the flexibility of the resulting hydrolyzates and the release of ionizable groups such as COOH and NH_2_ (Xu et al., 2021). Among hydrolyzates, H‐Pan and H‐Trp showed the highest WHC. In regard to the OHC, the highest value belonged to Alc‐H and Pep‐H. In another study, the OHC value of egg yolk hydrolyzates treated with Alcalase increased until the DH reached 10% and the excessive hydrolysis decreased this value (Bao et al., 2017).

### 3.4 Amino acid composition

As can be seen from the results, crude protein and its hydrolyzates were rich in acidic amino acids, especially glutamic and aspartic (Table 1). In general, hydrophobic amino acids (~34%) and antioxidants (~12.5%) constitute a significant part of the protein composition and hydrolyzates. The presence of high levels of HAAs, AAAs, and free types plays an important role in the reactivity of hydrolyzates with free radicals (Bozkurt et al., 2021). Samaei et al. conducted a study on the profile of hydrolyzates obtained from faba bean seed protein. In their study, methionine was considered as a limiting amino acid of faba bean. Moreover, the hydrolysis process had no significant effect on the content of most amino acids, but there was a significant increase in the histidine content in the pepsin hydrolyzate (Samaei et al., 2020). Also, in this study, the amount of essential amino acids (about 34%) and PER index (1.7–1.9) showed high nutritional quality and digestibility of protein and hydrolyzates. Similar results were reported in research conducted on *Spirulina* (Akbarbaglu et al., 2022) and Chinese beans (Ge et al., 2021) proteins.

**TABLE 1:** Amino acid composition of AKP and AKP hydrolyzates (mg amino acid/g dry sample).

Amino acid	Crude protein	AKPH‐Alc	AKPH‐Pan	AKPH‐Trp	AKPH‐Pep
Aspartic (Asp)	95.6	96.2	94.1	97.3	95.8
Glutamic (Glu)	210.5	206.5	211.4	213.7	211.3
Histidine (His)*^2^	24.2	24.9	24.8	25.1	24.4
Serine (Ser)	40.3	37.6	38.5	35.7	38.6
Arginine (Arg)	70.7	72.1	71.4	72.4	70.5
Glycine (Gly)	38.5	34.2	35.3	35.9	37.2
Threonine (Thr)*	20.4	21.4	21.2	22.5	21.6
Alanine (Ala)^1^	44.1	45.3	45.4	45.8	46.1
Tyrosine (Tyr)^12^	30.8	32.5	31.8	31.7	31.4
Methionine (Met)*^12^	4.8	5.2	5.8	5.2	5.3
Valine (Val)*^1^	36.3	38.5	37.2	37.4	36.9
Phenylalanine (Phe)*^1^	51.7	53.1	53.5	52.8	52.5
Isoleucine (Ile)*^1^	30.6	31.9	32.2	31.6	33.2
Leucine (leu)*^1^	57.4	59.2	55.9	58.8	59.1
Lysine (Lys)*^2^	24.1	26.1	25.2	25.3	24.6
Tryptophan (Trp)*^12^	11.3	12.7	11.5	11.9	12.2
NCAA	306.1	302.7	305.5	311.0	307.1
PCAA	119.0	123.1	121.4	122.8	119.5
HAA	267.0	278.4	273.3	275.2	276.7
AAA	95.2	101.4	99.1	99.2	97.9
EAA	260.8	273.0	267.3	270.6	269.8
EAA/TAA (%)	32.9	34.2	33.6	33.7	33.7
PER	1.82	1.86	1.74	1.87	1.89
TAA	791.3	797.4	795.2	803.1	800.7

Abbreviations: AKPH‐Alc, Apricot kernel protein hydrolyzed with Alcalase; Pan, Pancreatin; Pep, Pepsin; Trp, Trypsin.

*Essential amino acids (EAA); Negatively charged amino acids (NCAA) = asx (asparagine and aspartic acid) and glx (glutamine and glutamic acid); Positively charged amino acids (PCAA) = Arg, His, and Lys; ^1^Hydrophobic amino acids (HAA); ^2^Antioxidant amino acids (AAA); Total amino acids (TAA).

### 3.5 Antioxidant activity of hydrolyzates

The antioxidant activity of hydrolyzates is influenced by various factors, including molecular weight, DH, and amino acid composition. To assess the antioxidant capacity of hydrolyzates, different methods are employed, such as DPPH, ABTS, hydroxyl radical scavenging, reducing power, NO scavenging activity, total antioxidant capacity, and metal ion‐chelating activity.

#### 3.5.1 DPPH radical scavenging

**FIGURE 3:**
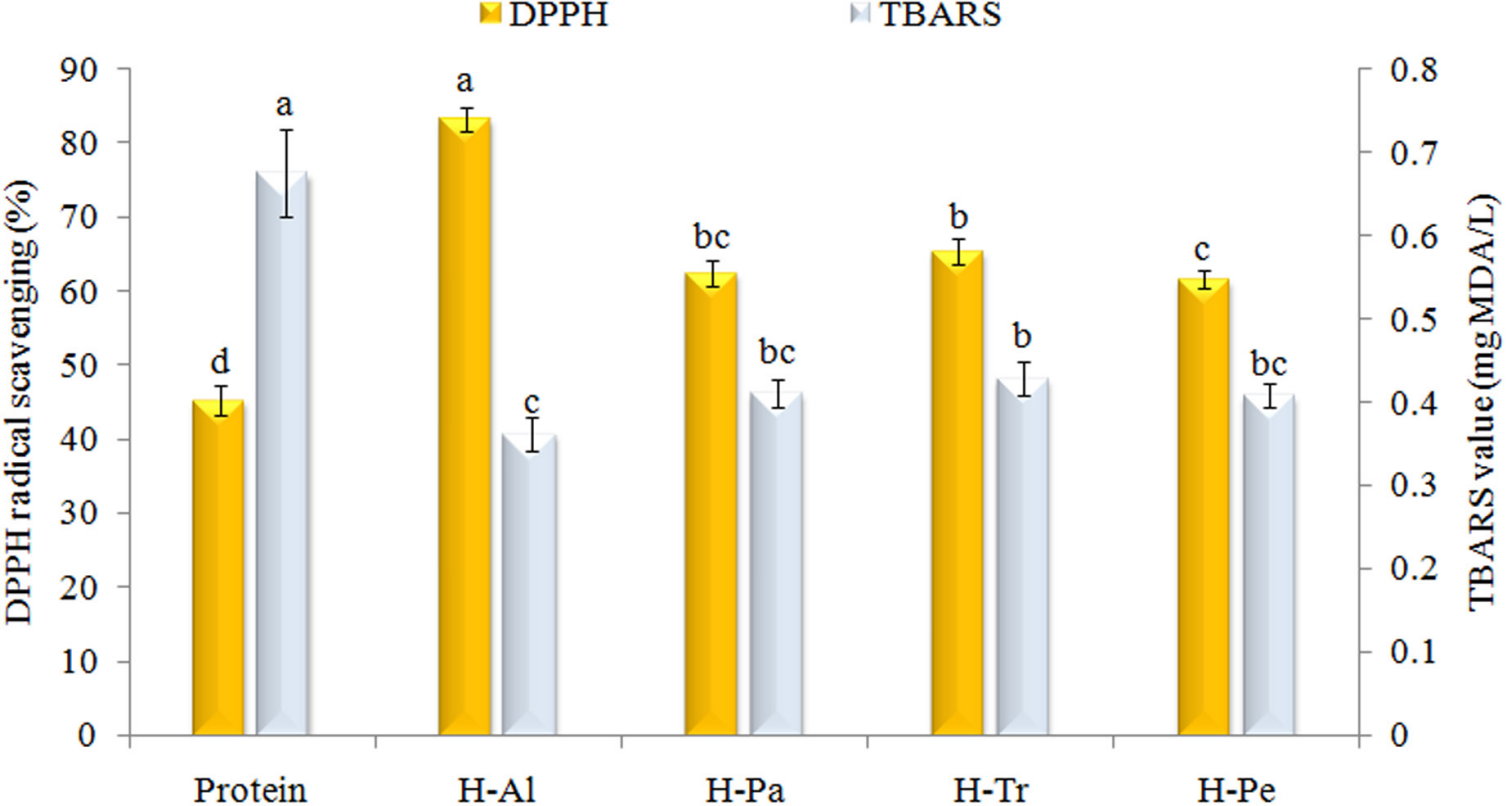
Effects of different enzymes on DPPH radical scavenging activity and TBARS value of apricot kernel protein. Al, Alcalase; H, hydrolyzate; Pa, pancreatin; Pe, pepsin; Tr, trypsin.

Figure 3 shows the DPPH radical scavenging of the AKP and its hydrolyzates. Al‐H (83.3%) exhibited a stronger ability to act as a hydrogen donor and scavenge free radicals. One possible explanation for this is that during Alcalase hydrolysis, the exposure of the protein substrate leads to the release of more hydrophobic amino residues. These hydrolyzates are then able to interact with lipid‐soluble DPPH radicals, making them more accessible for quenching the radicals effectively (Phongthai et al., 2018). Other hydrolyzates (H‐Pa, H‐Tr, and H‐Pe) showed the same trend which can show hydrolyzates with similar amino acid composition. In another study, the novel peptides isolated from grass carp skin using Alcalase enzyme showed excellent antioxidant activities (Cai et al., 2015).

It has also been observed that the DPPH scavenging capacity is positively associated with the presence of specific amino acids (Trp, Tyr, Met, Cys, His, and Phe) in the sequence of the hydrolyzates. hydrolyzates of thornback ray gelatin treated with proteolytic proteases from *Bacillus subtilis* A26 (TRGH‐A26), Raja clavata crude alkaline protease extract, Alcalase, and Neutrase were studied for their antioxidant activities by Lassoued et al. (2015). The hydrolyzates obtained by treatment with TRGH‐A26 showed the highest antioxidant activity as determined by the DPPH assay. This observation can be attributed to the hydrolyzates' elevated levels of His, Tyr, Met, and Phe, which surpassed those found in the other treatments (Lassoued et al., 2015).

#### 3.5.2 Oxidative stability in emulsion

Lipid oxidation is a critical factor that significantly impacts the quality attributes of various emulsified foods. The oxidation of unsaturated lipids not only leads to the development of unpleasant sensory characteristics but can also diminish the nutritional value and safety of the products. This is primarily due to the formation of primary and secondary oxidation products during processing and cooking, which can have adverse effects on the overall quality and stability of the food (Jang et al., 2016). To investigate the impact of the produced hydrolyzates on the oxidation stability of emulsions, the measurement of secondary oxidation products (TBARS value) was conducted in hydrolyzate‐coated emulsions. As shown in Figure 3, the TBARS value of the emulsions stabilized by crude protein, H‐Al, H‐Pe, H‐Pa, and H‐Tr were 0.7, 0.379, 0.419, 0.428, and 0.445 mg MDA/L in emulsions, respectively. The reasons for these findings can be attributed to the performance of antioxidant and hydrophobic peptides in preventing the formation of oxidation products and the production of TBARS in O/W emulsion (Zheng et al., 2019).

A similar result was achieved by other researchers. Mohammadi et al. (2022) reported that the amount of TBARS value in emulsions stabilized by *Spirulina* hydrolyzates treated with pepsin and pancreatin was lower than that of stabilized with *Spirulina* protein. Shahi et al. (2020) obtained a similar antioxidant activity trend in Alcalase and pancreatin‐treated protein. Liu et al. (2019) reported that the oxidation stability of O/W emulsion increased significantly by adding porcine bone hydrolyzates. In another study, the oxidation of the O/W emulsion was significantly inhibited by hydrolyzates treated with ficin, bromelain, or Alcalase, with the hydrolyzate obtained by bromelain showing the highest inhibition. This effect was attributed to the hydrolyzate's high hydrophobicity and emulsifying properties, as stated by the researchers. The combination of these factors likely contributed to the enhanced ability of the bromelain‐treated hydrolyzate to prevent oxidation in the emulsion (Zheng et al., 2019).

#### 3.5.3 ABTS radical scavenging

**FIGURE 4:**
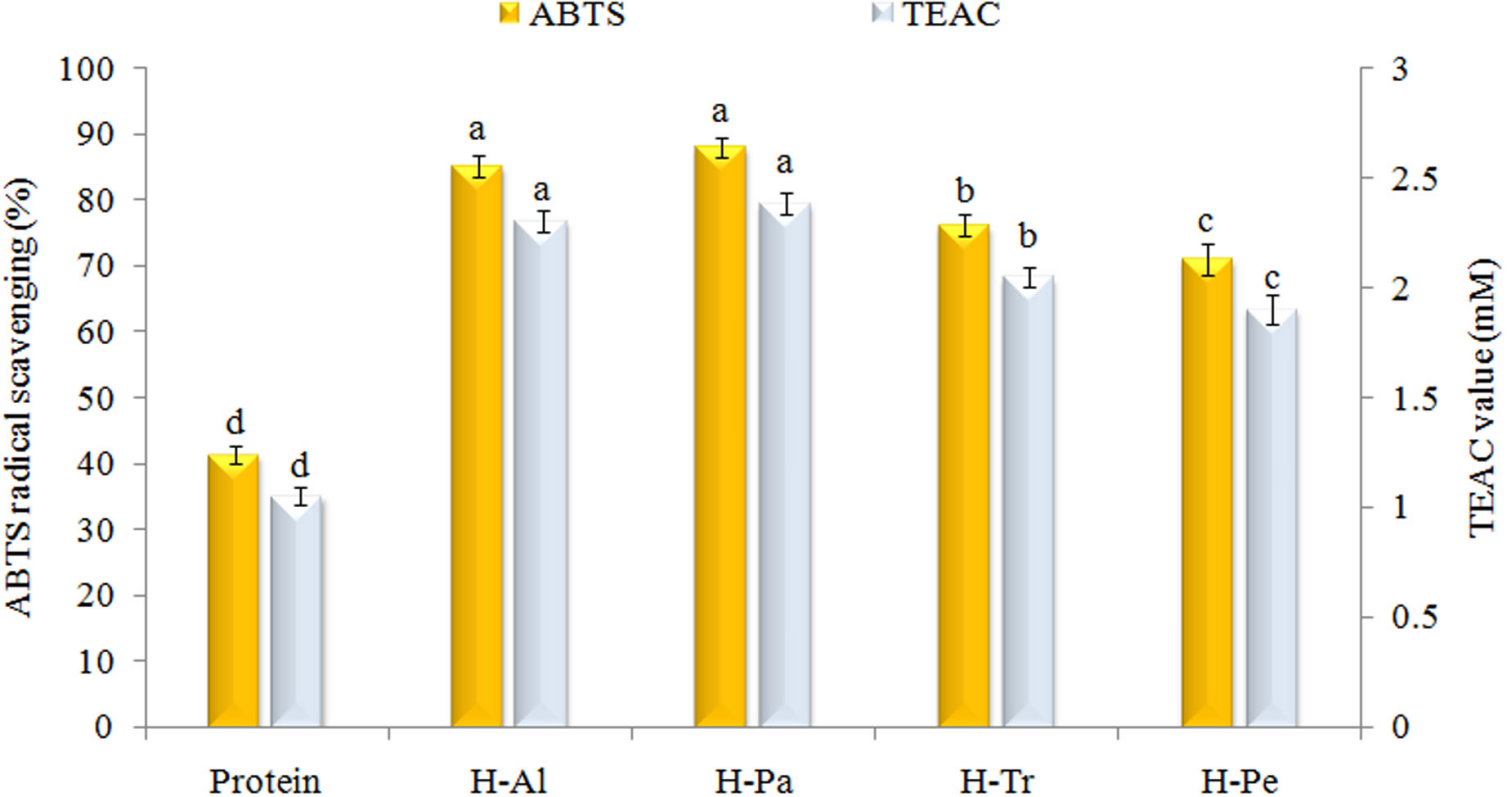
Effects of different enzymes on ABTS radical scavenging activity and TEAC value of apricot kernel protein. Al, Alcalase; H, hydrolyzate; Pa, pancreatin; Pe, pepsin; Tr, trypsin.

ABTS scavenging activity assesses the potential of both hydrophilic and lipophilic bioactive compounds for the elimination of ABTS radicals (Centenaro et al., 2014). In this assay, the bioactivity of the produced hydrolyzates is influenced not only by the type of enzyme used but also by the amino acid composition. Figure 4 demonstrates the ABTS radical scavenging activity of the various hydrolyzates and protein isolates. According to the data, the hydrolyzates exhibited varying levels of antioxidant activity, with H‐Pa (88.1%) and H‐Al (85.2%), demonstrating the highest activity, followed by H‐Tr (76.2%), H‐Pe (71.1%), and protein isolate (41.5%). The observed increase in antioxidant activity resulting from enzymatic hydrolysis can be attributed to the generation of peptides that possess the ability to donate electrons and hydrogen atoms. These peptides contribute to the inhibition of the radical, thereby enhancing the overall antioxidant capacity (Lima et al., 2019).

In a separate study investigating wheat glutenin hydrolyzates produced by different proteases, it was observed that the hydrolyzates treated with savinase and subsitilin, which had the lowest molecular weight, exhibited the highest ABTS radical scavenging activity. This observation could be attributed to the reduced steric hindrance of low‐molecular‐weight peptides (LMW) and the presence of hydrophobic and hydrophilic amino acid residues in the LMW sequence, which enhance their reactivity toward free radicals (Bozkurt et al., 2021). Particularly, the imidazole ring present in hydrophobic amino acid residues acts as an important proton donor, contributing to the high radical scavenging activity in oxidative reactions (Akbarbaglu et al., 2022). In another study, gluten hydrolyzates with relatively high hydrophobic features showed the highest ABTS scavenging activity (Lim et al., 2022). Moreover, for all the samples assessed, the antioxidant activity values were expressed as TEAC (mM Trolox equivalent per gram of sample; Figure 4).

#### 3.5.4 Hydroxyl radical scavenging

**FIGURE 5:**
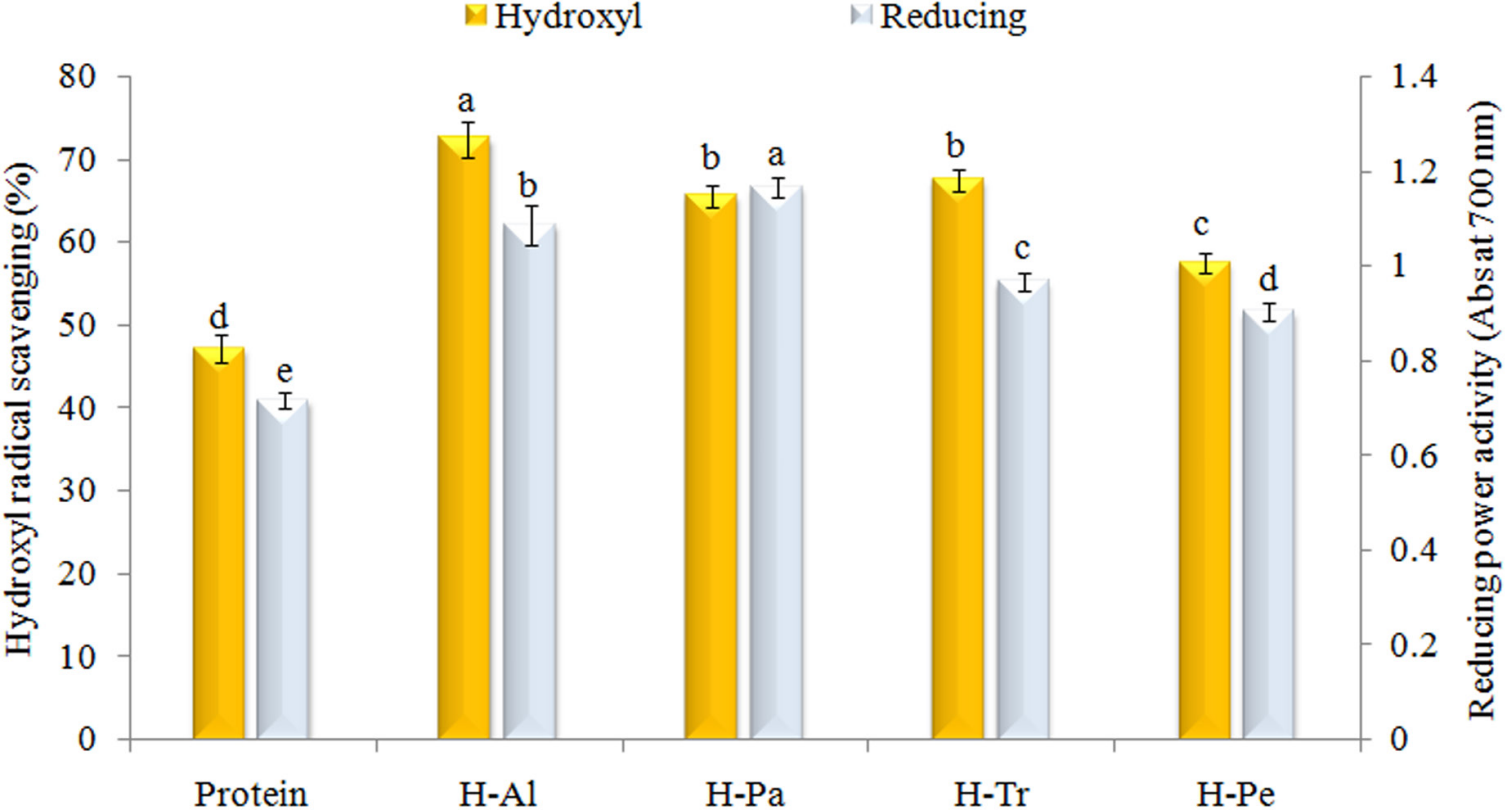
Effects of different enzymes on OH radical scavenging activity and reducing power of apricot kernel protein. Al, Alcalase; H, hydrolyzate; Pa, pancreatin; Pe, pepsin; Tr, trypsin.

In this study, the effects of different proteases on the inhibition of hydroxyl radical (as the initiator of lipid peroxidation and the destroyer of the biological systems) in AKP were investigated. As can be seen from Figure 5, enzymatic hydrolysis significantly increased the antioxidant activity of crude protein. Among the hydrolyzates, H‐Al showed a high capacity (72.5%) for scavenging hydroxyl radicals, and the H‐Pa and H‐Tr showed a similar trend (*p* < .05). The increase in antioxidant‐free amino acids, particularly aromatic amino acids such as histidine, tryptophan, phenylalanine, and tyrosine, resulting from enzymatic hydrolysis can be highlighted as one of the reasons for these observed findings (Moghadam et al., 2020).

In another study, it was found that walnut protein hydrolyzates exhibited notable hydroxyl radical scavenging activity, which was comparable to that of glutathione (Chen et al., 2012). Ambigaipalan et al. (2015) studied the enzymatic hydrolysis of date seed flour using Alcalase, Flavourzyme, and Thermolysin. The results of the study indicated that the hydrolyzates prepared using Alcalase exhibited the highest inhibition of hydroxyl radicals. On the other hand, the hydrolyzate produced by combining Alcalase and Thermolysin (Alcalase + Thermolysin) demonstrated the lowest inhibitory activity against hydroxyl radicals (Ambigaipalan et al., 2015).

#### 3.5.5 Reducing power

The total antioxidant activity (TAA), which reflects the electron‐donating ability of an antioxidant, can also be evaluated through the measurement of its reducing power. The reducing power of the produced hydrolyzates was presented in Figure 5. The H‐Pa and H‐Pe showed the highest (1.17) and the lowest (0.91) reducing power, respectively. The observed results can be attributed to the variations in the performance of enzymes in releasing antioxidant amino acids with electron or hydrogen‐donating ability, such as tryptophan, tyrosine, histidine, methionine, and lysine. These enzymes may exhibit different efficiencies in breaking down the protein structure and releasing these specific amino acids, which in turn affects the antioxidant activity of the hydrolyzates (Moghadam et al., 2020).

In a previous study, the reducing power of the pepsin‐treated walnut protein hydrolyzates had a dose‐dependent manner and this value was comparable to that of glutathione (Chen et al., 2012). In a separate study, the reducing power of date seed protein hydrolyzates treated with different enzymes (Alcalase, Flavourzyme, and Thermolysin) was examined. The results indicated that hydrolyzates prepared using Alcalase + Flavourzyme exhibited the highest reducing power. On the other hand, hydrolyzates obtained from Alcalase (AL) and Flavourzyme (FL) treatments demonstrated the lowest reducing power values (Ambigaipalan et al., 2015).

#### 3.5.6 NO scavenging activity

**FIGURE 6:**
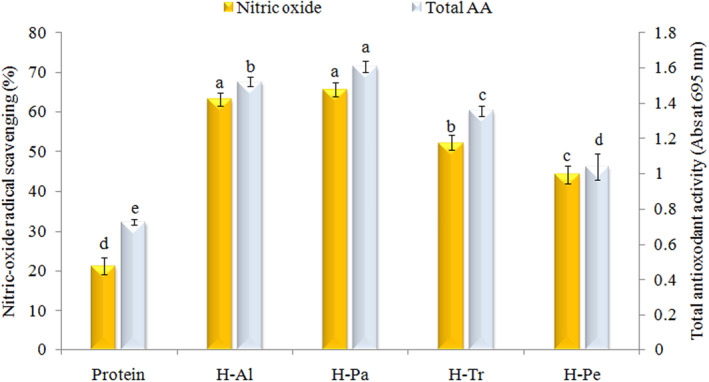
Effects of different enzymes on NO scavenging activity and total antioxidant activity of apricot kernel protein. Al, Alcalase; H, hydrolyzate; Pa, pancreatin; Pe, pepsin; Tr, trypsin.

The oxidation of L‐arginine to L‐citrulline results in the production of NO. Increased levels of NO have been associated with elevated risk factors for various conditions, including cancer, rheumatoid arthritis, atherosclerosis, and chronic inflammation (Akbarbaglu et al., 2019). Therefore, introducing natural antioxidants that can scavenge NO radicals can aid in keeping consumers healthy (López‐Barrios et al., 2016). According to Figure 6, it was observed that enzymatic hydrolysis increases the activity of protein in scavenging NO radicals by 2–3 times. The hydrolyzates H‐Pa (65.7%) and H‐Al (63.2%) exhibited the highest inhibition of NO radicals among the enzymes tested. It was reported that the existence of free amino acids such as aromatic types in the sequence of the hydrolyzates can be a significant factor for their NO radicals scavenging due to the hydrogen donor potential of hydroxyl groups of free amino acids for converting free radicals to more stable products (Kangsanant et al., 2015). In another study, a simultaneous combination of Alcalase and Flavourzyme enzymes increased the NO radicals scavenging ability of black bean hydrolyzates (Xu et al., 2021).

#### 3.5.7 Total antioxidant activity

The effect of in vitro hydrolysis on the antioxidant activity of the hydrolyzate samples was assessed by measuring their TAA, as depicted in Figure 6. The TAA values of the protein isolate notably increased with in vitro hydrolysis, following the trend: H‐Pa (1.61) > H‐Al (1.52) > H‐Try (1.35) > H‐Pep (1.1) > protein (0.73). This indicates that the hydrolysis process enhances the antioxidant activity of the protein, with H‐Pan demonstrating the highest TAA value among the tested hydrolyzates. The changing trend of the total antioxidant value may attribute to the different reaction mechanisms of this method compared to the other methods. According to Aguilar et al. (2019), the total hydrolyzed black bean protein exhibited an increase in antioxidant activity of approximately 31% compared to the non‐hydrolyzed sample. This increase in antioxidant activity was attributed to the hydrolysis process using a combination of Alcalase and Flavourzyme enzymes. In another research, the increase in TAA after enzymatic hydrolysis of flaxseed proteins (Sarabandi & Jafari, 2020a) and *Spirulina* (Akbarbaglu et al., 2022) with different proteases was reported.

#### 3.5.8 Metal ion‐chelating activity

**FIGURE 7:**
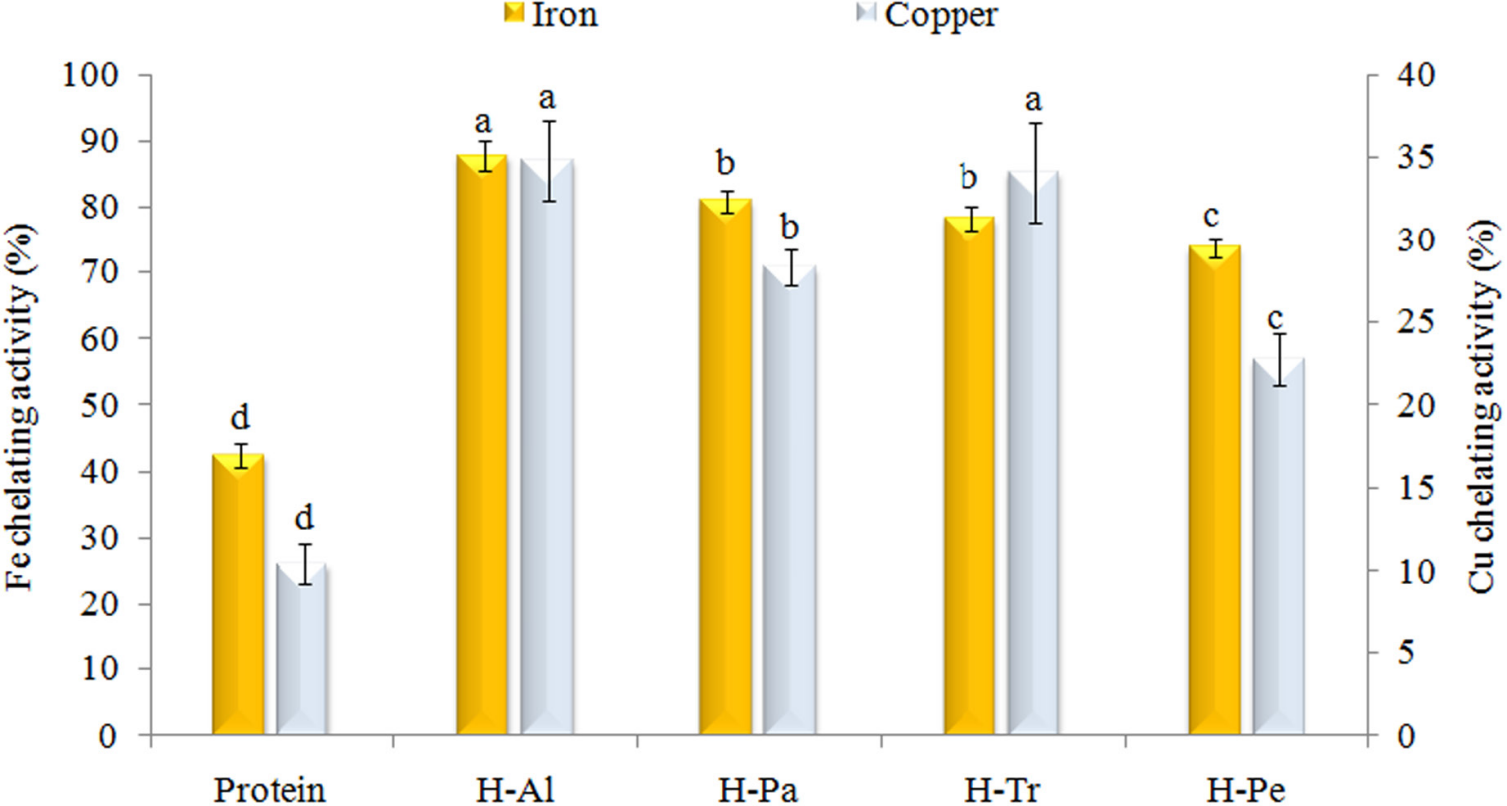
Effects of different enzymes on Fe^2+^‐ and Cu^2+^‐chelating activity of apricot kernel protein. Al, Alcalase; H, hydrolyzate; Pa, pancreatin; Pe, pepsin; Tr, trypsin.

Considering the rapid reaction between transition metal ions and peroxides, where the metal ions serve as one‐electron donors and generate alkoxyl radicals, we conducted measurements to evaluate the ability of apricot kernel hydrolyzates to donate electrons or hydrogen in order to reduce Fe^3+^ to Fe^2+^. The results showed that enzymatic hydrolysis significantly increased the chelation of metal ions in crude protein. As described in Figure 7, the Fe^2+^‐chelating capacity of the apricot kernel hydrolyzates showed the following order: H‐Al > H‐Pa and H‐Tr > H‐Pe > protein. The increase in free carboxyl (acidic amino acids) and amino (alkaline) groups, as well as the increase in ionic reactions as a result of enzymatic hydrolysis, can be considered as the potential reasons for these results (Islam et al., 2021). The chelating abilities of the different protein hydrolyzates have also been reported by several papers. Chen et al. (2012) stated that the chelating ability of the walnut protein hydrolyzates was found to be comparable to that achieved with ethylenediamine tetraacetic acid (EDTA) and significantly stronger than that of glutathione. In another study, antioxidant capacity was not directly correlated with the increased DH, as the hydrolyzates derived from Protamex with the lowest DH exhibited the highest antioxidant activity. In contrast, the hydrolyzates prepared with Alcalase showed varying antioxidant activities without a clear pattern of correlation with DH (Esfandi et al., 2019). In another study, there was a strong correlation between high amounts of arginine and lysine in sequences of hydrolyzates and their metal ion‐chelating activity (Torres‐Fuentes et al., 2012).

Cu^2+^‐chelating capacity of the apricot kernel hydrolyzates showed the following order: H‐Al > H‐Tr > H‐Pa > H‐Pe > protein. Recently, research has shown that low‐molecular‐weight hydrolyzates (105–1205 Da) and high contents of histidine amino acid (20%–30%) were correlated with high copper‐chelating activity (Torres‐Fuentes et al., 2012). Contrary to this report, in the case of phaseolin fractions, high‐molecular‐weight fractions exhibited a high copper‐chelating activity. Additionally, the fractions had low contents of Cys, Met, and His. The researchers proposed that the higher copper‐chelating activity in these fractions could be attributed to the high content of negatively charged amino acids (such as Asp and Glu) in their sequences (Carrasco‐Castilla et al., 2012).

### 3.6 Antimicrobial properties

The antimicrobial activity of the protein hydrolyzates has been reported in previous studies (Akbarbaglu et al., 2022; Da Rocha et al., 2018). Table 2 shows the inhibition zone of the AKP isolate and its hydrolyzates at a concentration of 50 mg/mL. A positive control sample of oxytetracycline solution (50 μg per well) was included. All samples showed inhibitory effects against the tested microorganisms (Gram‐negative: *E. coli O157:H7*; Gram‐positive: *B. cereus*), except for H‐Pan which did not show the inhibition effect against *B. cereus*. The hydrolyzates H‐Alc and H‐Pep demonstrated the highest inhibitory effects against *E. coli O157:H7*. For *B. cereus*, greater inhibition was observed with Trp hydrolyzates and pure protein. Interestingly, there was no clear correlation between the DH of the hydrolyzates and their antimicrobial effects against the microorganisms in this study. Da Rocha et al. (2018) reported that Alc hydrolyzates showed more inhibitory effects against *E. coli O157:H7* and *S. aureus* than those hydrolyzates obtained using Protamex with the same DH. The researchers proposed that the Alc hydrolyzates exhibited a higher proportion of hydrophobic amino acids, which could interact with the negatively charged regions on the bacterial surface through electrostatic interactions. This interaction may disrupt the bacterial cell wall and contribute to the observed antimicrobial effects (Da Rocha et al., 2018). Also, the presence of amino acids lysine and arginine increases the electrostatic attraction and reactivity of peptides with the cell membrane (Lima et al., 2019).

**TABLE 2:** Antimicrobial activity of apricot kernel protein (AKP) and AKP hydrolyzates.

Sample	Zone of inhibition (mm)	
	*Escherichia coli*	*Bacillus cereus*
OT	36.5 ± 0.6a	38.3 ± 0.6a
Protein	11.7 ± 0.8 cd	13.5 ± 0.7c
H‐Alc	16.1 ± 2.2b	10.1 ± 1.2e
H‐Pan	9.7 ± 1.5d	—
H‐Trp	12.6 ± 1.5c	15.4 ± 1.2b
H‐Pep	16.3 ± 0.6b	12.0 ± 1.1d

*Note*: Data are presented as mean ± standard deviation (*n* = 2) and different letters in the same column indicate significant differences at the 5% level in Duncan's test.

Abbreviations: Alc, Alcalase; H, hydrolyzate; OT, Oxytetracycline; Pan, pancreatin; Pep, pepsin; Try, trypsin.

## 4 CONCLUSION

The results of the study indicate that the hydrolyzates of AKP, particularly those treated with Alcalase, exhibited strong scavenging activity against various radicals (DPPH, ABTS, OH, and NO) compared to the crude protein. Additionally, these hydrolyzates displayed notable reducing power, metal ion‐chelating activity, and TAA. The amino acid composition, essential amino acid ratio, and protein efficiency ratio index suggested that the apricot kernel hydrolyzates have good nutritional quality and high digestibility. Furthermore, the peptides derived from the enzymatic hydrolysis of AKP demonstrated effective antibacterial properties against *E. coli* and *B. cereus*. Based on these findings, it is suggested that antioxidant hydrolyzates derived from AKP have the potential to serve as natural antioxidants and preservatives in the production of functional protein supplements.

## AUTHOR CONTRIBUTIONS

**Khashayar Sarabandi:** Data curation (equal); project administration (equal); supervision (equal); writing – review and editing (equal). **Maryam Mohammadi:** Investigation (equal); methodology (equal); writing – original draft (equal). **Zahra Akbarbaglu:** Conceptualization (equal); investigation (equal); methodology (equal); resources (equal). **Marjan Ghorbani:** Methodology (equal); writing – review and editing (equal). **Shahla Najafi:** Data curation (equal); writing – review and editing (equal). **Sara Safaeian Laein:** Investigation (equal); methodology (equal); resources (equal). **Seid Mahdi Jafari:** Data curation (equal); supervision (equal); writing – review and editing (equal).

## CONFLICT OF INTEREST STATEMENT

The authors confirm that they have no conflicts of interest with respect to the work described in this manuscript.

## ETHICS STATEMENT

Ethics approval was not required for this research.

## Data Availability

Data are available on request to the corresponding author.
